# Pan-AMPK activator O304 prevents gene expression changes and remobilisation of histone marks in islets of diet-induced obese mice

**DOI:** 10.1038/s41598-021-03567-3

**Published:** 2021-12-23

**Authors:** Ana López-Pérez, Stefan Norlin, Pär Steneberg, Silvia Remeseiro, Helena Edlund, Andreas Hörnblad

**Affiliations:** 1 Umeå Centre for Molecular Medicine (UCMM), Umeå University, Johan Bures väg 12, 90187 Umeå, Sweden; 2 Wallenberg Centre for Molecular Medicine (WCMM), Umeå University, 90187 Umeå, Sweden

**Keywords:** Transcription, Metabolic syndrome, Pre-diabetes, Type 2 diabetes, Genomics, Gene regulation

## Abstract

AMP-activated protein kinase (AMPK) has an important role in cellular energy homeostasis and has emerged as a promising target for treatment of Type 2 Diabetes (T2D) due to its beneficial effects on insulin sensitivity and glucose homeostasis. O304 is a pan-AMPK activator that has been shown to improve glucose homeostasis in both mouse models of diabetes and in human T2D subjects. Here, we describe the genome-wide transcriptional profile and chromatin landscape of pancreatic islets following O304 treatment of mice fed high-fat diet (HFD). O304 largely prevented genome-wide gene expression changes associated with HFD feeding in CBA mice and these changes were associated with remodelling of active and repressive chromatin marks. In particular, the increased expression of the β-cell stress marker *Aldh1a3* in islets from HFD-mice is completely abrogated following O304 treatment, which is accompanied by loss of active chromatin marks in the promoter as well as distant non-coding regions upstream of the *Aldh1a3* gene. Moreover, O304 treatment restored dysfunctional glucose homeostasis as well as expression of key markers associated with β-cell function in mice with already established obesity. Our findings provide preclinical evidence that O304 is a promising therapeutic compound not only for T2D remission but also for restoration of β-cell function following remission of T2D diabetes.

## Introduction

The current global epidemic of Type 2 Diabetes (T2D) poses a major health threat and is strongly associated with the worldwide rise in obesity^1^. Exercise and controlled diets improve insulin sensitivity and are the first treatment recommendations for T2D patients, but long-term compliance is difficult and for some patients exercise and diet does not suffice or are not feasible options. Notably, accumulating data show that, provided that β-cell function has not deteriorated too far^2^, remission of T2D with restoration of β-cell function can be achieved following a very low calorie diet^3^, intensive insulin therapy^4^ and bariatric surgery^5^. However, how remission of T2D diabetes, e.g. following caloric restriction, affects the β-cell on a molecular level is largely unknown. A key mediator of the effects of exercise and diet/caloric restriction on glucose metabolism is AMP-activated protein kinase (AMPK)^6^. AMPK, the key sensor of cellular energy homeostasis, is activated under conditions of low energy and phosphorylates targets in numerous signalling pathways, including lipid homeostasis, mitochondrial biogenesis, and glycolysis, thus favouring catabolic processes at the expense of anabolism^7^. Given its important role in cellular energy homeostasis and positive effects on insulin sensitivity, AMPK has emerged as a promising target for treatment of T2D.

We recently described a novel pan-AMPK activator, O304, that exerts beneficial effects on glucose homeostasis and vascular health both in high-fat diet (HFD) induced obese mice and in human T2D subjects^8^. Importantly, O304 treatment preserved β-cell function and secretory capacity by alleviating metabolic stress, largely through increased glucose uptake in the peripheral tissues thus improving insulin sensitivity and consequently promoting β-cell rest^8^. Notably, direct effects of O304 in alleviating ER stress and inhibiting amyloid formation in β-cells were also demonstrated in ex vivo cultured islets^8^. The β-cell molecular consequences following O304 mediated in vivo restoration of glucose homeostasis are however not fully understood. In particular, genome-wide changes in the transcriptional profile of pancreatic islets, as well as potential changes in genome-wide epigenetic chromatin marks, have not been assessed in the context of O304-mediated remission of insulin resistance and dysfunctional glucose homeostasis. To study the effects of O304 mediated amelioration of insulin resistance and dysglycemia on the transcriptional landscape and epigenetic modifications in pancreatic islets within an in vivo prediabetic context, we here describe the analyses of transcriptomes as well as chromatin marks in pancreatic islets of O304 treated HFD-mice. We show that O304 treatment largely prevented islet gene expression changes associated with HFD-induced obesity in pancreatic islets and preserved the distribution of epigenetic signatures associated with active or repressed chromatin. Furthermore, O304 treatment restored glucose homeostasis and gene expression levels of key markers involved in β-cell stress in islets of HFD-induced obese mice.

## Results

### O304 treatment prevents islet gene expression signature changes induced by HFD

We have previously demonstrated that the AMPK activator O304 improves blood glucose homeostasis in both human T2D subjects as well as in high-fat diet induced obese and diabetic mouse models. In the present study, we have now analysed the in vivo effects of O304 treatment on the genome-wide transcriptional program of the pancreatic islets. To characterize gene expression signatures and chromatin marks of β-cells in the context of elevated glucose levels induced by obesity, and the potential positive effects of O304-mediated remission of dysglycemia in this context, we performed RNA-sequencing and Chromatin ImmunoPrecipitation sequencing (ChIP-seq) of islets isolated from 20-week-old mice on regular diet (RD), high-fat diet (HFD) and HFD formulated with O304 (HFD-O304). Diets were introduced at 10 weeks of age. Fasting blood glucose and plasma insulin levels were similar between the groups at start of the diet (Supplementary Fig. S1a). In line with our previous data^8^, HFD-O304 mice had significantly lower glucose and insulin levels than HFD mice after 9 weeks of the treatment period (Supplementary Fig. S1b). Food intake was highest for RD mice (4.2 g/m/day) and lowest for HFD mice (2.5 g/m/day), whereas food intake was increased in HFD-O304 mice compared to HFD fed mice (3.2 g/m/day, Supplementary Fig. S1c). A blunted first-phase insulin release is the first detectable defect in β-cell function in T2D aetiology and can be assessed by arginine stimulated insulin secretion tests, which provides an estimate of the β-cell secretory capacity independent of potential increased peripheral glucose uptake/improved insulin. Arginine stimulated insulin secretion was improved in HFD-O304 mice as compared to non-treated HFD mice (Supplementary Fig. S1d), confirming previous data on improved first-phase insulin secretory capacity of the β-cells in O304 treated diet-induced obese mice^8^.

**Figure 1:**
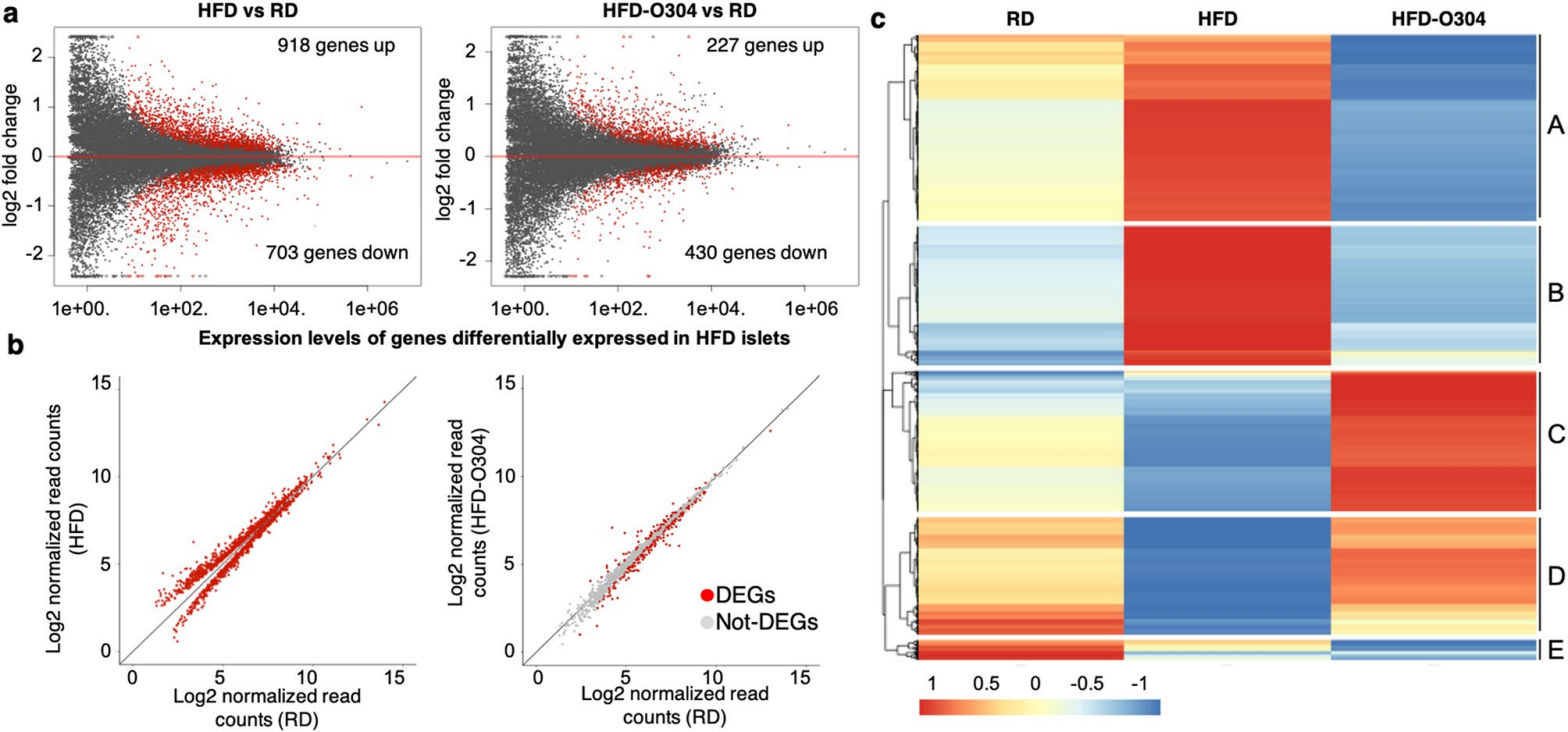
O304 treatment prevents islet gene expression changes induced by HFD in mice. (**a**) MA plot of gene expression in HFD islets versus RD islets (left) and HFD-O304 islets versus RD-islets (right). Red dots indicate differentially expressed genes (FDR < 0.01). (**b**) Gene expression levels of differentially expressed genes between HFD islets and RD islets (left) and the same genes plotted between HFD-O304 islets and RD islets (right). Red dots indicate genes that are differentially expressed in each condition (FDR < 0.01). Note that the vast majority of genes differentially expressed in HFD islets are expressed at normal levels in HFD-O304 islets. (**c**) K-means clustering of genes differentially expressed between RD (left), HFD (middle), and HFD-O304 islets (right). Scale from red to blue indicates fold change over average read count for each row.

RNA-seq and ChIP-seq analysis of islets isolated from mice following 10 weeks of treatment demonstrated that 1621 genes were differentially regulated in islets of HFD mice compared with that of RD mice (918 upregulated, 703 downregulated: FDR < 0.01) (Fig. 1a, left, Supplementary Table S1). In contrast, islets isolated from HFD-O304 mice presented a transcriptional profile similar to islets of RD-mice, with only 657 differentially expressed genes between islets of these two groups of mice (227 upregulated, 430 downregulated) (Fig. 1a, right, Supplementary Table S1). Of the 1621 differentially expressed genes in HFD mice, 1495 corresponds to genes that are not differentially expressed between islets of HFD-O304 mice and RD controls (Fig. 1b and Supplementary Fig. S2a). This implies that O304 prevented, directly or indirectly, the dysregulation of the majority of genes whose expression is altered in this HFD provoked pre-diabetic context. In fact, O304 preserved the expression of > 90% of the differentially expressed genes in islets of HFD mice (Fig. 1b). Accordingly, both principal component analysis (PCA) (Supplementary Fig. S2b) and hierarchical clustering (Supplementary Fig. S2c) group HFD-O304 islet samples closer to islets from RD controls than to islets of HFD mice. Taken together, these findings provide evidence that O304 treatment prevents HFD induced islet transcriptional changes, thus largely preserving transcriptional profiles similar to islets of RD mice.

K-means clustering analysis of differentially expressed genes revealed 4 major clusters, out of a total of 5, where clusters A and B contain genes primarily upregulated in islets of HFD mice, and clusters C and D contain genes primarily downregulated in islets from HFD mice (Fig. 1c). Enrichment analysis of gene ontology (GO) terms and KEGG pathways revealed profound functional differences between the expression profiles of the three experimental groups (Supplementary Fig. S3 and Supplementary Table S1). The most enriched GO- and KEGG-terms for cluster A (containing genes that were upregulated in HFD islets) are related to endoplasmatic reticulum (ER) function and protein processing, including response to ER stress (Supplementary Fig. S3, Supplementary Table S1). This finding corroborates our previous data on ex vivo cultured islets where O304 treatment prevents increased expression of genes related to unfolded protein response under high-glucose conditions^8^. In the setting of obesity-provoked insulin resistance, β-cell proliferation is a compensatory response to the increased demand of insulin. Consistently, upregulation of genes in cluster B, which are mainly related to cell cycle and proliferation (Supplementary Fig. S3, Supplementary Table S1), were observed in islets of HFD mice, but not in islets of HFD-O304 mice. β-Cell proliferation has also been suggested to be tightly coupled to a loss of mature β-cell identity^21^, and AMPK has been implicated in maintenance of a mature β-cell phenotype via inhibition of mTORC1 signalling^22^. Still, while β-cell proliferative markers were not induced in islets of HFD-O304 mice (Supplementary Fig. S4a), we find no clear evidence for altered mTORC1 signalling (Supplementary Fig. S4b). Taken together these findings show that O304 treatment prevents up-regulation of genes associated with metabolic β-cell stress. However, given that O304 averts HFD induced insulin resistance^8^ (Supplementary Fig. S1), the effect of O304 on β-cell stress is likely partly indirect.

Genes in Cluster C, which were most abundantly expressed in islets of HFD-O304 mice and least expressed in HFD mice, were strongly enriched for terms/pathways related to regulation of hormonal secretion and insulin secretion in particular (Supplementary Fig. S3, Supplementary Table S1), as well as genes related to AMPK- and glucagon-signalling (Supplementary Table S1). Compared to islets from RD mice, O304 treatment preserved or even increased the expression of these genes in mice fed HFD (Supplementary Fig. S3, Supplementary Table S1). These findings suggest that, in the context of HFD, O304 treatment enhances the insulin secretory capacity of β-cells by increasing the abundance of transcripts related to these processes. Cluster D was enriched in genes largely related to neuronal function, which may reflect that β-cells share several phenotypic traits with neurons that are dependent on similar transcriptional programs for their function^23^. Last, cluster E genes are mainly associated with amino acid metabolism and other catabolic processes (Supplementary Fig. S3), likely reflecting that AMPK activation inhibits protein synthesis and stimulates catabolic processes^24^.

Taken together, these data demonstrate that O304 treatment largely prevented the transcriptional changes observed in islets of HFD mice. Although the net action of O304 treatment on β-cells likely occurs indirectly via amelioration of insulin resistance and dysglycemia as well as through a combination of direct downstream targets, it significantly impacts on several key β-cell processes affected in T2D aetiology, and in particular O304 treatment prevented gene expression changes associated with insulin signalling and hormonal secretion.

### O304 treatment mitigates gene expression signatures associated with β-cell stress

To further understand the impact of O304 treatment on glucose homeostasis and β-cell function in the context of HFD and insulin resistance, we explored our transcriptional dataset to investigate the effects on specific genes relevant for β-cell function and/or β-cell identity, and whose altered expression has been implicated in β-cell stress. As HFD mice are not overt diabetic but exhibit a pre-diabetic glucose intolerant phenotype, islets of HFD-mice were expected to display subtle expression changes, primarily of early markers of β-cell stress. Preserved expression levels of such markers would imply a maintained β-cell phenotype, and the functional annotation of differentially expressed genes (Supplementary Fig. S3, Supplementary Table S2) in our data indeed suggest that this may be the case.

**Figure 2:**
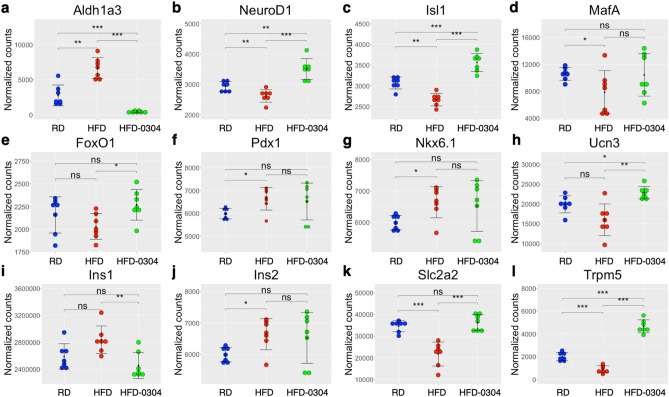
O304 treatment improve islet gene expression signatures associated with β-cell stress and impaired insulin secretion. Dotplots of normalized read counts for β-cell stress genes in islets from RD controls (blue), HFD mice (red) and HFD-0304 mice (green) (n = 7 for all groups) Individual data points, mean ± SEM are indicated. The Wilcoxon rank sum test was applied. *ns* not significant, *p < 0.05, **p < 0.01, ***p < 0.001.

Aldehyde dehydrogenase 1 isoform A3 (ALDH1A3) is a proposed marker for metabolically stressed β-cells^25–29^ and in agreement with these findings, *Aldh1a3* expression was significantly increased in islets from HFD-mice compared to RD controls (Fig. 2a). Notably, *Aldh1a3* expression was reduced in islets of HFD-O304 mice compared with both HFD and RD islets (Fig. 2a). Increased *Aldh1a3* expression in β-cells has been linked to reduced expression of key transcription factors associated with maintenance of β-cell identity and function^27^. No major differences in gene expression were observed for a set of mature β-cell identity genes although the expression profile of HFD-O304 islets were more similar to RD islets than to HFD islets^22,30^ (Supplementary Fig. S5a). However, O304 treatment not only prevented the reduced expression of *NeuroD1* and *Isl1* (Fig. 2b,c) observed in HFD islets but also enhanced the expression of these genes compared with that observed in RD islets (Fig. 2b,c). In addition, a similar trend was observed for *MafA,* the expression of which was slightly decreased in HFD islets (Fig. 2d) as compared to RD and HFD-O304 islets. *FoxO1* that regulates *NeuroD1* and *MafA* was slightly upregulated in HFD-O304 islets while O304 treatment had no effect on *Pdx1* or *Nkx6.1* expression (Fig. 2e–g)*.* The limited transcriptional changes observed for only a subset of β-cell identity genes suggest that increased *Aldh1a3* expression is not directly linked to a reprogrammed β-cell state but rather appear to be a general marker of early metabolic β-cell stress.

*Urocortin-3*, *Ucn3*, is a marker for functionally mature β-cells that showed reduced expression under metabolic stress conditions^31,32^. *Ucn3* expression tended to be decreased in HFD islets as compared with that of RD islets and O304 significantly increased *Ucn3* expression compared with that of both RD and HFD islets (Fig. 2h). Taken together, these findings indicate that preservation of β-cell function in O304 treated HFD mice involves both maintenance of mature β-cell identity and amelioration of β-cell stress.

Reprogramming due to metabolic stress has also been associated with increased expression of “disallowed genes”, i.e. genes normally repressed in β-cells^33–36^. Although we do not observe major changes in the expression of such genes, the transcriptional profile of this gene set also tends to be more similar between RD islets and HFD-O304 islets (Supplementary Fig. S5b). This finding suggests that the expression of disallowed genes in dysfunctional β-cells is limited at very early stages of T2D but gradually increases in response to sustained hyperglycemia and progression of T2D.

### O304 treatment preserves the expression of insulin secretory associated genes in HFD mice

HFD mice displayed highly increased levels of fasting plasma insulin (Supplementary Fig. S1a), and islet mRNA levels of *Ins1* tended to be elevated while the abundance of *Ins2* transcripts were significantly increased in HFD compared to RD islets (Fig. 2i,j). In HFD-O304 mice, *Ins2* transcripts still tended to be increased compared to RD islets whereas *Ins1* mRNA levels were significantly reduced as compared to HFD mice (Supplementary Fig. S1a and Fig. 2i), likely reflecting a decreased demand for insulin production and secretion as a consequence of improved insulin sensitivity. The lower metabolic load on β-cells of HFD-O304 mice would in turn contribute to β-cell rest and preserved β-cell function. Interestingly, the differentially expressed genes in our data were enriched for GO-terms associated with response to Glucose Stimulus and Insulin Secretion (GSIS) (Supplementary Fig. S3, Supplementary Table S1). Hierarchical clustering of samples based on genes related to insulin secretion (99 genes from 3 gene sets) segregates HFD islets from RD and HFD-O304 islets, which further underscores the preservation of gene expression profiles related to insulin secretion in HFD-O304 islets as compared to lean controls (Supplementary Fig. S6).

Under conditions of hyperglycemia and perturbed GSIS, the glucose transporter *Slc2a2* is markedly downregulated, which correlates with an impaired insulin secretory response in both mice and rats^37–40^. Consistently, *Slc2a2* expression was downregulated in HFD islets as compared to RD islets, and this expression was completely restored in islets of HFD-O304 mice (Fig. 2k). We observed a similar expression pattern for *Trpm5*, another glucose-responsive gene also associated with functional GSIS^41,42^. Alike *Slc2a2*, HFD resulted in downregulation of *Trpm5* expression in islets (Fig. 2l) suggesting that reduced levels of *Trpm5* is also an early indicative of β-cell stress and disturbed glucose homeostasis. However, in HFD-O304 islets, *Trpm5* levels were increased compared with both HFD and RD islets (Fig. 2l). These data demonstrate that, in pre-diabetic HFD mice, O304 treatment largely normalized islet gene expression signatures associated with β-cell stress and impaired glucose response. Taken together, these findings provide evidence of preserved β-cell health status in HFD mice treated with O304 that in turn might prevent long-term functional deterioration of β-cells due to overnutrition.

### O304 treatment induces remobilisation of chromatin marks in islets of HFD mice

**Figure 3:**
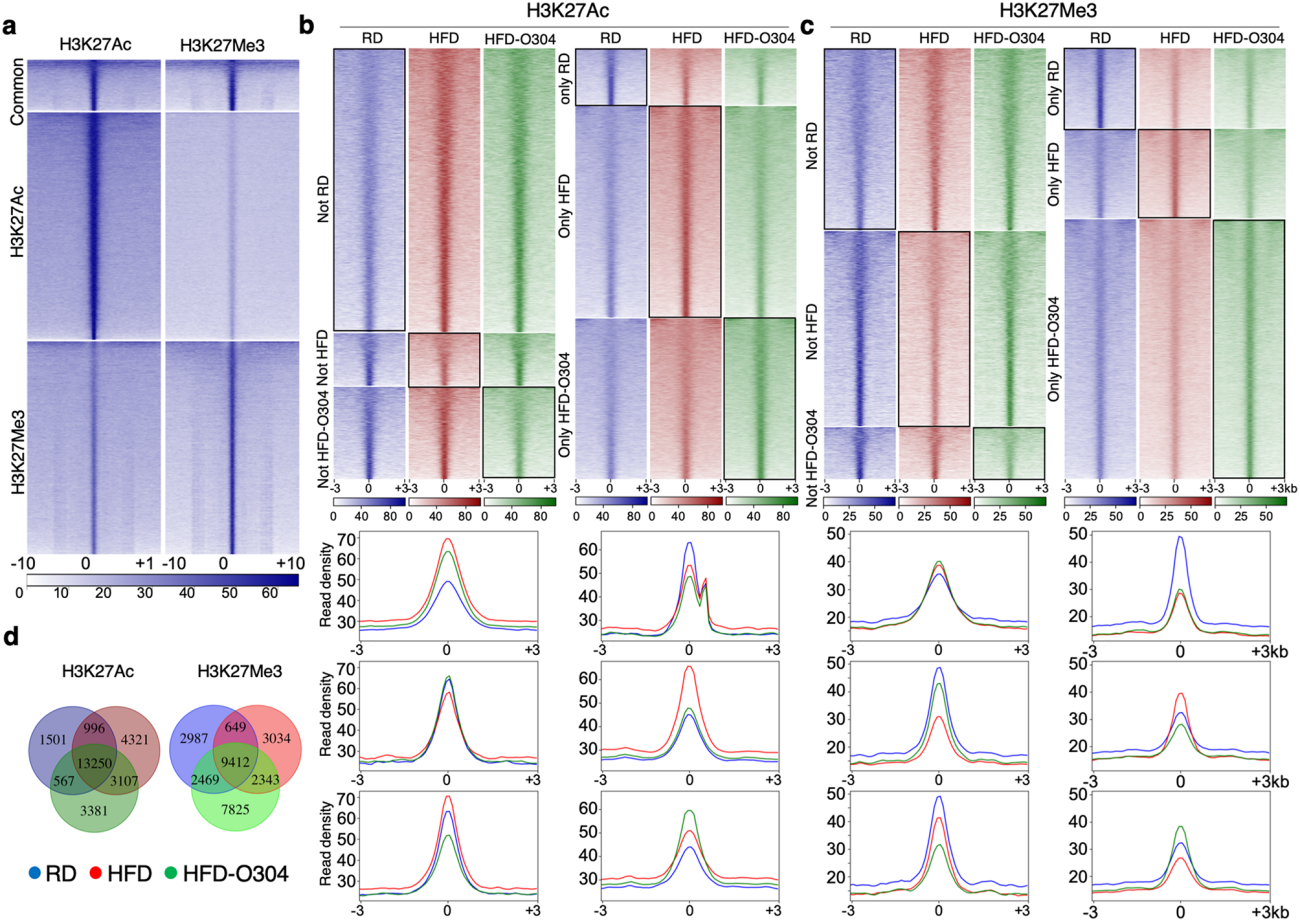
O304 treatment induces chromatin remodelling in pancreatic islets of HFD mice. (**a**) Heatmaps of read distribution in a ± 10 kb window of called ChIP-seq peaks for H3K27Ac and H3K27Me3 in islets from RD control mice. Colorbar below heatmaps indicate ChIP-seq read count. (**b**,**c**) Upper panel: heatmaps of read distribution in a ± 3 kb window for (**b**) H3K27Ac and **(c)** H3K27Me3 ChIP-seq peaks. Lower panel: density plots of read distribution across ChIP-seq peaks displayed in the heatmaps. (**d**) Venn diagram showing the overlap of H3K27Ac (left) and H3K27Me3 (right) peaks for RD, HFD, and HFD-O304 islets. (FDR < 0.01).

In order to further elucidate how O304 impacts on gene regulation and epigenetic marks, we characterized the distribution of active (H3K27Ac) and repressive (H3K27Me3) chromatin marks by ChIP-seq in islets from RD controls, HFD, and HFD-O304 mice. In RD islets we identified 16,314 H3K27Ac and 15,517 H3K27Me3 binding regions which present a highly non-overlapping distribution as expected (Fig. 3a and Supplementary Fig. S7a). We also observed that active and repressive chromatin marks correlated positively or negatively, respectively, with gene expression, further validating the quality of our data (Supplementary Fig. S7b,c).

A large fraction of the called peaks for both active (H3K27Ac) and repressed (H3K27Me3) chromatin were common for RD, HFD, and HFD-O304 islets. Importantly, a substantial number of peaks were unique for each condition or only present in two of them (Fig. 3b–d). Interestingly, the abundance of H3K27Ac binding sites was higher in HFD and HFD-O304 islets, as compared to RD islets (21,674; 20,305; and 16,314 respectively). In HFD islets, binding of H3K27Ac to 7428 new genomic regions, that were not present in RD islets, was observed together with the depletion of this mark from 2068 sites as compared with that of RD islets (Fig. 3b,d). O304 treatment prevented H3K27Ac binding in 4321 out of these 7428 new sites and also preserved its binding to 567 of the 2068 sites lost in HFD islets (Fig. 3b,d). These results provide evidence that O304 prevented ~ 58% of the redistribution of active chromatin marks observed in HFD islets, as well as preserved ~ 27% of H3K27Ac regions lost in HFD islets (Fig. 3d).

In contrast to H3K27Ac, the number of H3K27Me3-enriched regions detected in islets from lean controls and HFD mice were similar, while these regions were more abundant in HFD-O304 islets (15,517; 15,438; and 22,049 respectively). Still, the distribution of these regions was considerably different, where only 9412 of the peaks were shared between the three groups (Fig. 3c,d). In HFD islets, 5377 new H3K27Me3-enriched regions were detected as compared to RD, but > 55% (3034) of these did not carry H3K27Me3 in HFD-O304 islets (Fig. 3d). In addition, 45% (2469 out of the 5456) of regions depleted for H3K27Me3 in HFD islets as compared to RD islets were preserved in islets of HFD-O304 mice (Fig. 3d). Taken together, these findings suggest that O304 treatment also prevented the redistribution of a large fraction of repressive chromatin marks induced by HFD in islets.

Interestingly, most of the active and repressed regions that were preserved by O304 treatment were located in intergenic regions, not in promoters (< 1 kb from TSS), suggesting that at least some of the gene expression changes induced in HFD mice and preserved in HFD-O304 mice may depend on regulatory input from distal enhancer elements (Supplementary Fig. S7). These data provide evidence that remobilisation of active and repressive chromatin marks contributed to the observed gene expression changes associated with HFD, and that O304 treatment preserved a significant fraction of the epigenetic signature characteristic of lean RD islets.

### Increased *Aldh1a3* gene expression is accompanied by changes in histone modifications

**Figure 4:**
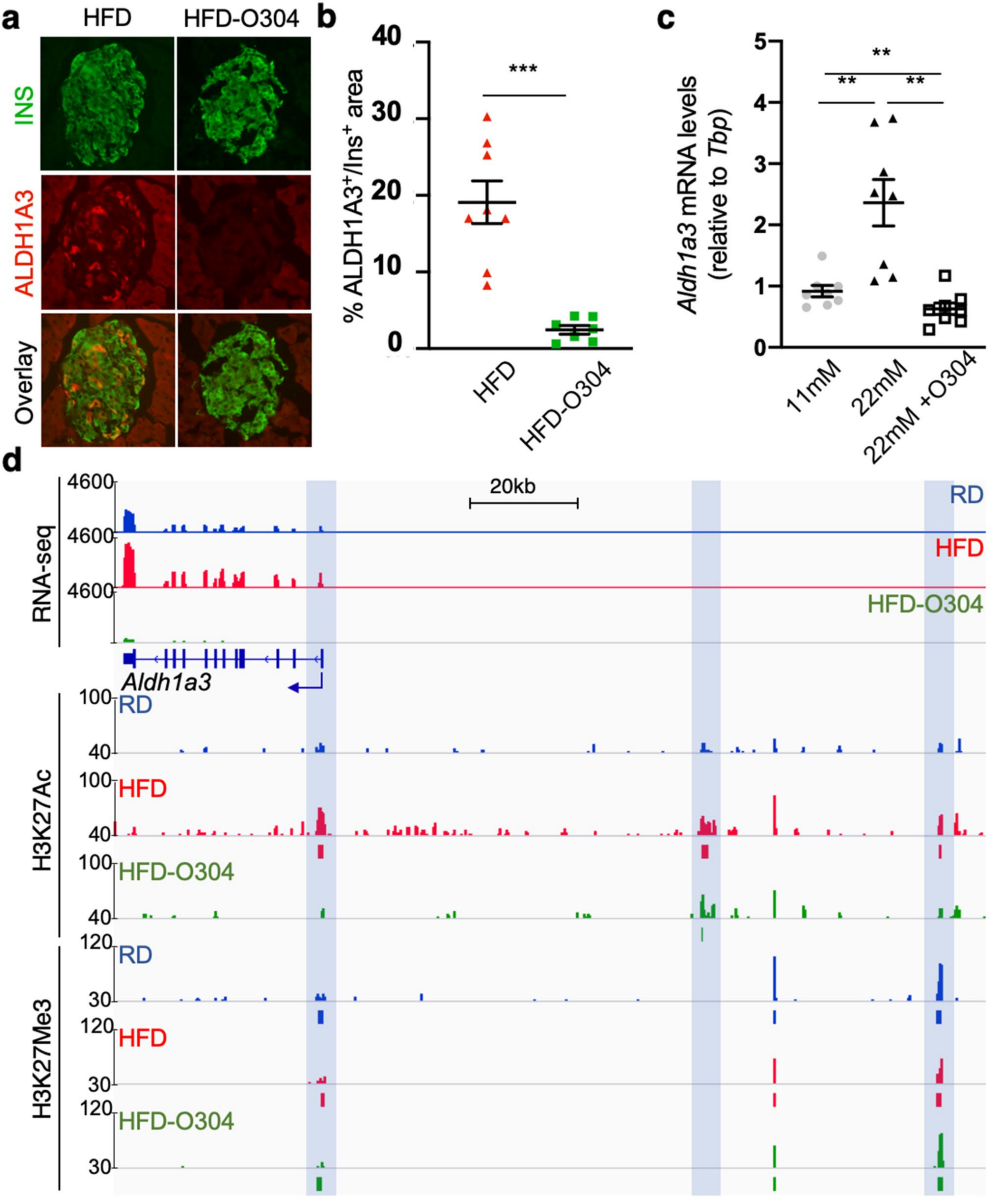
O304 mediated downregulation of *Aldh1a3* is accompanied by redistribution of H3K27Ac. (**a**) Representative sections of HFD and HFD-O304 pancreata stained for insulin (green) and ALDH1A3 (red). (**b**) Quantification of the proportion of insulin positive area with immunoreactivity for ALDH1A3 in HFD (n = 8) and HFD-O304 (n = 7). ***p < 0.001 (two-tailed Student’s *t* test). Individual data points, mean ± SEM are indicated in all graphs. (**c**) Relative mRNA expression of *Aldh1a3* in CBA islets cultured in vitro under conditions of low glucose (11 mM), high glucose (22 mM), and high glucose (22 mM) plus O304 (5 μM) (n = 7 for all conditions) **p < 0.01. (Wilcoxon signed rank test). (**d**) Read count in the *Aldh1a3* locus for RNA-seq (upper panel), H3K27Ac ChIP-seq (middle panel) and H3K27Me3 ChIP-seq (lower panel) in RD (blue), HFD (red) and HFD-O304 (green) islets. Called peaks are indicated as boxes below read count. Blue shading indicates areas of differential acetylation.

As stated above, *Aldh1a3* has in recent years emerged as a potential biomarker for dysfunctional β-cells^27^, and *Aldh1a3* expression is significantly increased in our RNA-seq data in HFD islets, but not in treated HFD-O304 islets. In addition, a significant decrease in the percentage of ALDH1A3-positive β-cell area in HFD-O304 mice (~ 2.5%) with respect to that of HFD mice (~ 19%) (Fig. 4b,c) was observed. Notably, in isolated islets cultured ex vivo* Aldh1a3* expression was significantly increased at high glucose concentrations (22 mM), as compared with islets cultured at 11 mM glucose concentration, whereas *Aldh1a3* expression in islets cultured at 22 mM glucose in the presence of 5 μM O304 was significantly reduced even compared with that of islets cultured at 11 mM glucose (Fig. 4a). Thus, O304 prevented an increase in *Aldh1a3* expression both in vivo in islets of HFD-O304 mice and ex vivo in isolated islets cultured at high glucose levels. Together these data provide evidence that O304 ameliorates β-cell stress both directly and indirectly, i.e. via reduced metabolic burden.

To investigate further how these changes in gene and protein levels correlated with regulatory changes, we investigated H3K27Ac and H3K27Me3 occupancy at the *Aldh1a3* locus in our ChIP-seq data. Notably, the increased expression of *Aldh1a3* in HFD-islets was accompanied by enrichment of H3K27Ac in the promoter region as well as two distant regions (~ 70 kb and ~ 114 kb) upstream of the gene (Fig. 4d). None of these binding sites were present in islets of RD mice, and in HFD-O304 islets both the promoter region and the most distant element were devoid of H3K27Ac-enrichment. In addition, in islets from RD controls, HFD or HFD-O304 mice, all of these regions were bound by H3K27Me3, likely reflecting a heterogenous β-cell population with only a subset (~ 19%, Fig. 4b,c) of the β-cells of HFD mice expressing ALDH1A3. Most importantly, these findings also provide evidence that *Aldh1a3* expression is actively repressed via H3K27Me3 in healthy islets and, as a consequence of the increased metabolic pressure induced by HFD, expression of *Aldh1a3* is activated upon remobilisation of H3K27Ac to the gene promoter and to two distal upstream elements that may represent potential regulatory regions*.* Taken together, these data demonstrate that O304 treatment induced remobilisation of chromatin marks in pre-diabetic islets, which thus likely contributes to preservation of their transcriptional profile.

### O304 treatment reverts gene expression changes associated with β-cell stress and impaired insulin secretion

**Figure 5:**
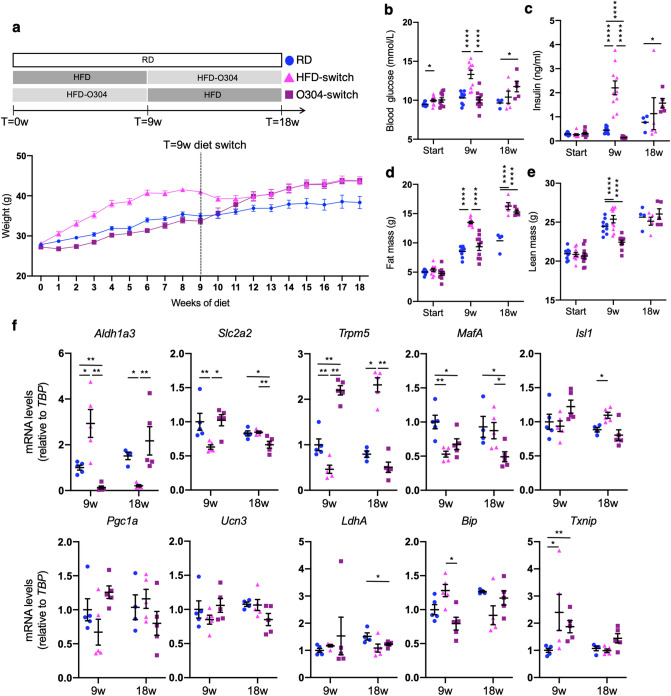
O304 treatment reversed impaired glucose homeostasis in HFD mice. (**a**) Weight curve for RD (blue circles), HFD-switch (pink triangles) and O304-switch (purple squares). Dashed line indicates the timepoint for switch of diet. Fasted blood glucose (**b**) and insulin (**c**), fat mass (**d**) and lean mass (**e**) for the same mice at start of diet, at 9 weeks when diet switch was performed and at 18 weeks (from start until 9 weeks n = 9 for RD, 10 for HFD-switch, and 10 for O304-switch, thereafter 5 mice from each group were sacrificed and n = 4 for RD, 5 for HFD-switch, and 5 for O304-switch at 18 weeks). Mean ± SEM is indicated for weight curve while all other graphs display individual data points, mean ± SEM. (**f**) Relative mRNA expression of key islet genes in RD, HFD-switch and O304-switch islets. Significant expression changes as compared to RD controls in each of the time points are displayed with asterisks. *p < 0.05, **p < 0.01, ****p < 0.0001 (Wilcoxon rank sum test).

To test whether O304 treatment could not only prevent, but also revert the islet gene expression changes associated with diet-induced obesity, mice were fed HFD for 9 weeks and then switched to HFD formulated with O304 (0.8 mg/g) for another 9 weeks (from here on referred to as HFD-switch mice) (Fig. 5a). Vice versa, mice that had been fed HFD-O304 for 9 weeks were switched to HFD without O304 (from here on referred to as O304-switch mice). Control mice were fed RD throughout the whole time period. Mice fed HFD had a significantly decreased food intake (> 30% reduction) as compared to both mice fed RD or HFD-0304, but food intake increased after the switch to HFD-O304 (Supplementary Fig. S8). Reciprocally, food intake of HFD-O304 mice was reduced after the switch to HFD (Supplementary Fig. S8).

In HFD-switch mice the transition from HFD to HFD-O304 completely reverted the hyperinsulinemia, dysglycemia, and insulin resistance established by the 9 weeks HFD feeding (Fig. 5b,c). Notably, although weight gain was halted by switching mice from HFD to HFD-O304, neither body weight, lean body mass, nor fat mass decreased after the switch from HFD only to HFD-O304 (Fig. 5d,e), suggesting that the positive metabolic effects exerted by O304 treatment are not secondary to weight loss. In O304-switch mice, before the switch (T = 9 weeks), fasting plasma insulin were slightly decreased and glucose levels similar compared with that of RD mice (Fig. 5a–c). The switch from HFD-O304 to HFD resulted in an increased weight and fat mass gain as well as a marked increase in insulin and glucose levels (Fig. 5b–e). Together, these data demonstrate that O304 treatment not only prevents dysglycemia in HFD mice but also potently reverses dysfunctional glucose homeostasis in pre-diabetic HFD mice, and that this is uncoupled from changes in body mass.

To test if the improved metabolic profile of HFD-switch mice is also accompanied by significant improvements in islet gene expression profiles, we isolated total RNA from pancreatic islets at the time of the switch (Fig. 5a, T = 9 weeks) as well as after 9 weeks of intervention (Fig. 5a, T = 18) and analysed the expression of key islet genes associated with β-cell stress and insulin secretion. Importantly, HFD-switch mice presented with an improved gene expression profile at T = 18 weeks for key islet genes associated with β-cell stress and insulin secretion (Fig. 5f), indicating that O304 treatment can revert gene expression changes associated with HFD. The expression of the β-cell stress marker *Aldh1a3* was reduced to minimal levels in HFD-switch islets after 9 weeks of treatment, while both *Slc2a2* as well as *Trpm5* expression was restored, and in the latter case even increased as compared to controls (Fig. 5f). Together these data provide evidence that O304 treatment potently can reduce β-cell stress and improve glucose sensing and secretory capacity also after an initial 9-week period of HFD feeding. The expression of β-cell identity gene *MafA* was restored in HFD-switch islets at T = 18 weeks and *Isl1* expression was significantly increased as compared to control islets (Fig. 5f). The trend was similar for the mitochondrial biogenesis regulator *Pgc1a* (Fig. 5f). In addition*,* in line with previous ex vivo data on islets cultured in high glucose^8^, at T = 18 weeks the expression of the unfolded protein response genes *Bip* and *Txnip* were reduced, providing further support that O304 treatment reverted β-cell stress in HFD-switch mice. In O304-switch islets, the opposite change in expression profiles was observed with increased expression of *Aldh1a3*, as well as a decreased expression of *Slc2a2*, *Trpm5*, and *MafA* at T = 18 weeks, in comparison to the timepoint of the switch, i.e. T = 9 weeks (Fig. 5f)*.* We did not detect any major change in the expression levels of the β-cell maturity marker *Ucn3* nor in that of the disallowed gene *Ldha* at T = 18 weeks for HFD-switch islets. Taken together, these data demonstrate that O304 treatment not only potently prevents impaired glucose homeostasis and islet transcriptional changes associated with high-caloric food intake in HFD mice, but that O304 treatment also can restore metabolic function and associated islet gene expression changes in HFD mice with established obesity and dysglycemia.

## Discussion

The potential of AMPK as a target for treatment of metabolic disease, including T2D, has received much attention in recent years. Still, no novel drugs developed to directly target AMPK have reached the market. We recently described the pan-AMPK activator O304 and its beneficial effects on whole body glucose homeostasis, cardiovascular health and islet function in mice and humans^8^. Here, we demonstrate that O304 treatment not only improves the transcriptional status of certain genes under conditions of in vivo metabolic stress but also potently prevents the global transcriptional changes associated with diet-induced obesity in islets of mice. Moreover, as shown by our diet-switch experiments, O304 treatment reverted HFD induced dysglycemia in mice, which was also accompanied by a downregulation of β-cell stress gene expression and an improved transcriptional profile as compared to that of islets from non-treated HFD mice. Interestingly, some of the gene expression changes induced by O304 treatment in vivo is recapitulated in islets cultured ex vivo, indicating that O304 may positively impact β-cell function via two different mechanisms: indirectly by reducing the metabolic load on the β-cells through improved glucose uptake in the peripheral tissues, and directly by relieving β-cell stress responses and preserving a functional β-cell identity. Although it appears likely that indirect effects (i.e. improved insulin sensitivity) exert a major role in the improved in vivo gene expression profiles, that does not exclude that O304 also exerts direct effects on β-cells, which will be the focus of future studies.

In T2D, numerous mechanisms (e.g. glucotoxicity, insulin resistance, oxidative and ER-stress) have been identified to contribute to the gradual decline of β-cell function^43^. However, although the molecular mechanisms remain largely unknown, alleviation of metabolic stress has been associated with restoration of β-cell function^3–5^. Under conditions of sustained metabolic stress, β-cells lose their functional identity, which is manifested as a loss of functional β-cell mass. If metabolic stress perseveres, β-cells may progress to apoptosis, eventually leading to true β-cells loss at later stages of T2D development^44^. Loss of β-cell identity refers to the process whereby mature β-cells to various degrees lose their glucose stimulated insulin secretion capacity, downregulate expression of genes ensuring β-cell function, and upregulate expression of genes that are disallowed in the normal physiological context^33,34,36,44–46^. In islets of HFD fed mice, we did not observe clear changes in β-cell identity genes. Nonetheless, it is striking that islets of HFD-O304 mice not only cluster closer to islets of RD mice in terms of whole transcriptome profiles but also in analyses based on groups of genes involved in specific processes related to β-cell function. Together, these findings imply that deterioration of β-cell identity in T2D may not arise concomitantly with functional decline but rather develop secondary to loss of β-cell function. This is also in concordance with the idea that the cellular state of a reprogrammed β-cell is dependent on the diabetogenic context rather than due to reversal of developmental ontogeny^47^. Given the growing number of reports of increased expression of disallowed genes in challenged β-cells^33^, one could speculate that small stochastic transcriptional changes are amplified in the setting of metabolic stress, and that additional cofactors (e.g. genetic, environmental) in the long term potentiate “transcriptional drift” and subsequent downregulation of genes key for maintenance of functional β-cell identity, thus eventually leading to the development of overt T2D.

Insulin secretion and underlying gene regulation is tightly coupled to nutrient state^45,46^ and adaptive insulin secretion has been suggested to be controlled through epigenetic changes^46^. In support of this notion, our data show that the transcriptional changes induced by high-fat diet, and the prevention of these changes by O304 treatment, are accompanied by the redistribution of active and repressive chromatin modifications. Although analysis of islets isolated from HFD mice showed that the increased mRNA expression of the early β-cell stress marker *Aldh1a3* was accompanied by increased H3K27Ac in the promoter region and at upstream intergenic regions, immunohistochemical analyses revealed ALDH1A3 upregulation only in a subset of β-cells. In view of this limitation in our study, as well as the increasing evidence of β-cell subpopulations and differences in cellular states, an important open question is how the detected global transcriptional and epigenetic changes translates to cellular heterogeneity in this context.

In summary, our findings demonstrate a remarkable positive effect of O304 in vivo treatment on islet gene expression signatures in the context of HFD induced insulin resistance and dysglycemia. Taken together, our pre-clinical findings provide evidence that O304 holds great therapeutic potential for future clinical use for prevention and restoration of β-cell failure in T2D subjects.

## Methods

### Animals and diets

All experiments were performed in compliance with national and institutional laws and guidelines and the study is reported in accordance with ARRIVE guidelines. The study was approved by the Regional Ethics Committee at the Court of Appeal of Northern Norrland. Ethical approval ID A-4-2019. CBA males were chosen for this study as they, in contrast to C57Bl/6 mice, do not display inherent impaired insulin secretion. CBA mice also respond well to high-fat diet and readily develop insulin resistance and hyperinsulinemia^8^, mimicking the recently defined human SIRD diabetes subtype^9^. For ex vivo culture of islets CBA x C57Bl/6 F1 hybrid mice were used. Mice were purchased from Charles River Lab and housed at 12:12 h light/dark cycle in a temperature/humidity controlled (22 °C and 50% humidity) room and *ad libitum* feeding with either standard chow (Special Diet Service #801730), high-fat diet (HFD) (Research diets, Inc. #D12492) or HFD supplemented with 0.8 mg/g O304 (HFD-O304) (Research diets, Inc. #D12492 custom formulated with 0.8 mg/g O304 (kindly provided by Betagenon AB)). 10-weeks-old CBA mice were allocated to standard chow, HFD, and O304-HFD groups for 9 weeks. After 9 weeks mice were either sacrificed or switched from HFD to HFD-O304, and vice versa, for an additional 9 weeks. Food intake and body weight were measured weekly. Due to the different colour of the experimental diets, investigators were not blinded to the group allocation of experimental animals.

### Metabolic measurements and body composition

Body composition of live mice was measured using EchoMRI (EchoMRI LLC; EchoMRI 3-in-1). Arginine stimulated insulin secretion tests were performed on 6-h fasted mice under sedation with Hypnorm (Veta Pharma)/Midazolam (Hamlenmice) followed by intraperitoneal (i.p.) administration of arginine (1 g/kg body weight; SIGMA #A5131). Blood glucose levels were measured using Glucometer (Ultra 2, One Touch) and plasma insulin was analysed via the ultrasensitive mouse insulin ELISA kit (Chrystal Chem Inc. #90080).

### Islet preparation

Islets were isolated by collagenase digestion of the pancreas as previously described^10^. Briefly, mice were killed by cervical dislocation and the pancreata were perfused with 2.5 ml cold collagenase (without Mg^2+/^Ca^2+^, GIBCO #14180). The pancreata were then dissected out and incubated at 37 °C for 10–15 min. Collagenase activity was stopped by adding 25 ml of Hanks’ solution (with Mg^2+/^Ca^2+^, GIBCO #14060) and vigorous shaking released the islets from the rest of the pancreatic tissue. Islets were rinsed in ice-cold Hanks’ three times and then picked manually under a stereo microscope.

### RNA-seq

Isolated islets from 5 mice were pooled (n = 7 for all conditions) and 150 islets were used for extraction of total RNA islets using RNeasy Micro Kit (Qiagen, #74004). RNA-seq libraries were prepared with the Illumina TruSeq RNA Library Kit according to the manufacturer’s instructions. 50 bp paired-end sequencing was performed on a NovaSeq 6000 Illumina Sequencer at The National Genomics Infrastructure (SciLifeLab -Science for Life Laboratory, Stockholm), with coverage of ~ 40 million reads per library.

For RNA-seq analyses, Fastq files with 50-nt paired-end sequenced reads were quality-checked with FastQC (https://www.bioinformatics.babraham.ac.uk/projects/fastqc/) and raw reads were aligned to the mouse genome (GRCm38, Ensembl genome version 96, ensembl.org) using STAR (options: *--outSAMtype BAM SortedByCoordinate --seedSearchStartLmax 12 --outFilterScoreMinOverLread 0.3 --alignSJoverhangMin 15 --outFilterMismatchNmax 33 --outFilterMatchNminOverLread 0 --outFilterType BySJout --outSAMattributes NH HI AS NM MD --outSAMstrandField intronMotif --quantMode GeneCounts*)^11^.

Genes with a minimum row sum of 10 reads were kept for further analysis. Normalization and differential expression analysis were performed using DESeq2 (p < 0.01 and False Discovery Rate (FDR) < 0.01)^12^. Plotting of individual gene expression was done based on the normalised read counts obtained in DESeq2 and Wilcoxon test was applied for mean comparison.

Clustering of differentially expressed genes was obtained using pheatmap package. Gene Ontology (GO) and KEGG enrichment analysis for pairwise comparisons and specific cluster were performed using clusterProfiler (p < 0.01 and FDR < 0.01)^13^.

In order to visualise the GO terms containing differentially expressed genes that best classify the three predefines samples, GOexpress package was used (Bioconductor, Rue-Albrecht, 2020). The plotting script was modified to visualise the genes present in the customised terms.

### ChIP-seq

For ChIP-seq experiments ~ 600 islets were processed (n = 2 for each condition). Chromatin immunoprecipitation (ChIP) was performed as previously described with some modifications^14^. Briefly, islets were fixed 15 min in 1 ml fixing solution (1% formaldehyde, 50 mM HEPES–KOH, 100 mM NaCl, 1 mM ethylenediaminetetraacetic acid (EDTA), 0.5 mM EGTA) at RT with gentle mixing. Crosslinking was stopped by adding 1/20 volume of 2.5 M glycine for 5 min at RT. Islets were then washed 2 × 5 min at 4 °C with cold 1 × PBS containing protease inhibitor cocktail (Roche, #4693132001), snap frozen and stored at − 80 °C until awaiting further processing. Pooled islets were lysed 5–10 min in 130 μl of lysis buffer at 4 °C and sonicated on a Covaris E220 device (PIP: 105, Duty factor: 2, Cycles/burst: 200, Duration: 9 min). ChIP was performed with rabbit anti-H3K27Ac (Abcam, ab4729) and rabbit anti-H3K27Me3 (Upstate #07-449) antibodies. ChIP-seq libraries were prepared with the Rubicon Thruplex DNA-Seq kit following manufacturer’s instructions library preparations and sequencing were performed. 50 bp paired-end sequencing was performed on a NovaSeq 6000 Illumina Sequencer, at the National Genomics Infrastructure (SciLifeLab -Science for Life Laboratory, Stockholm), with coverage of ~ 60 million reads per library.

Fastq files with 50-nt paired-end sequenced reads were quality-checked with FastQC (https://www.bioinformatics.babraham.ac.uk/projects/fastqc/) and raw reads were mapped to the mouse genome (GRCm38, Ensembl genome version 96, ensembl.org) using bowtie2 (options: *--threads 4 --very-sensitive*)^15^*.* Samtools was used to convert SAM files into BAM files and the BAM files for each replicate were merged. Enriched peaks (p < 0.01, FDR < 0.01) were called using MACS2 (options: *callpeak --gsize* 1.87e9 *--*broad *--*broad-cutoff 0.1 -f BAMPE -q 0.1 *--*nolambda *--*nomodel *--*shift 200 --extsize 200)^16^. findOverlapsOfPeaks function from ChIPpeakAnno package was used to define the whole set of peaks for each histone modification and to define if a peak was common or specific for each condition^17,18^.

To study the correlation of ChIP-seq and RNA-seq a Student’s *t* test was performed to compare the read counts in RNA-seq for each gene and the binding at transcription starting site (TSS) of the histone marks determined by ChIP-seq. Heatmaps and profile plots of scores around genomic regions were performed with deepTools^19^ and annotation of the peaks was done with ChIPseeker^20^.

For signal profiles and correlation patterns plots, we calculated the histone mark signal for all genes in each of the 200 bins that we split the 6000 bp surrounded the TSS. For visualization, the average signal across all genes was calculated and then normalized against the maximum value in these 200 bins to always have a maximum value of one. The correlation pattern was obtained by correlating the signal in each bin with the gene expression levels using the Spearman correlation coefficient for each of the bins.

### Ex vivo culture of islets

For ex vivo analyses, islets were cultured for 96 h in RPMI medium 1640 (GIBCO #11879-0) supplemented with 11.1 or 22.2 mM glucose (GIBCO #A24940-01), 1% fetal bovine serum (GIBCO #10500), 50 U/ug Pen/Strep per ml (Gibco #15140-122), 10 mM Hepes (Umeå University, Laboratory medicine), 1 mM sodium pyruvate (GIBCO #11360-039) and 0.1% 2-Mercaptoethanol (Sigma#M3148). 5 μM O304 were added from day 0 of culture; the control contained DMSO 1:2000.

### qRT-PCR

Total RNA was prepared from isolated islets using RNeasy Micro Kit (Qiagen #74004) and first strand cDNA synthesis was done using Superscript III (Invitrogen #18080-051). qPCR was performed with FastStart Universal SYBR Green master (rox) (Sigma-Aldrich # 4913850001) on a Bio-Rad CFX Connect instrument. Data were normalized using TBP as the reference gene. Primers used for qPCR are listed in Supplementary Table S3.

### Immunofluorescence and morphometric analysis of islets

Quantification of β-cells expressing ALDH1A3 was done on cryosections from pancreatic tissues isolated from HFD (n = 8 mice, a total of 90 islets) and HFD-0304 mice (n = 7 mice, a total of 75 islets) kept on diet for 9 weeks. Isolated pancreata were fixed 1.5 h in 4% PFA, and prepared for cryosectioning. Immunostainings were performed using as primary antibodies α-ALDH1A3 (1:1000, Novus Biologicals NBP2-15339) and α-Ins (1:500, Dako A0564), and Alexa Fluor 488 (1:500, Jackson ImmunoResearch 706-545-148) and Alexa Flour-594 (1:500, Invitrogen a-11012) as secondary antibodies. Quantification of insulin and ALDH1A3 positive area was determined using Fiji software (Version 2.0.0).

### Statistical analysis

Sample-size estimates were not performed prior to the execution of the study and no data points were excluded. Prism 9 was used for statistical analysis of body composition, metabolic parameters, imaging data and qRT-PCR data. Normality and lognormality tests were performed to select appropriate statistical methods for analysis. RNA-seq and ChIP-seq data were analyzed using specific R-packages (see separate section).

## Supplementary Information

Supplementary Information.

Supplementary Table S1.

Supplementary Table S2.

Supplementary Table S3.

## Data Availability

All generated datasets for RNA-seq and ChIP-seq are available through the Gene Expression Omnibus repository under the accession number GSE190813.
