# Supplementary material for: Pan-AMPK activator O304 prevents gene expression changes and remobilisation of histone marks in islets of diet-induced obese mice
Source: Sci Rep. 2021 Dec 23;11:24410. doi: 10.1038/s41598-021-03567-3 (PMC8702551; doi:10.1038/s41598-021-03567-3)
Supplement: Supplementary file 1 — Supplementary Information. [file 41598_2021_3567_MOESM1_ESM.pdf]

Pan-AMPK activator O304 prevents gene expression changes and remobilisation of histone marks in islets of diet-induced obese mice.

Ana López-Pérez[1], Stefan Norlin[1], Pär Steneberg[1], Silvia Remeseiro[1, 2], Helena Edlund[1, *] & Andreas Hörnblad[1, *]

1. **Umeå Centre for Molecular Medicine (UCMM), Umeå University, Johan Bures väg 12, 90187 Umeå Sweden**
2. **Wallenberg Centre for Molecular Medicine (WCMM), Umeå University, 90187 Umeå, Sweden**

* **Corresponding authors: A.H (Tel. +46705574988, andreas.hornblad@umu.se) and H.E (Tel. +46706739986 helena.edlund@umu.se)**

Author e-mail:

ana.lopez@umu.se

stefan.norlin@umu.se

par.steneberg@umu.se

silvia.remeseiro@umu.se

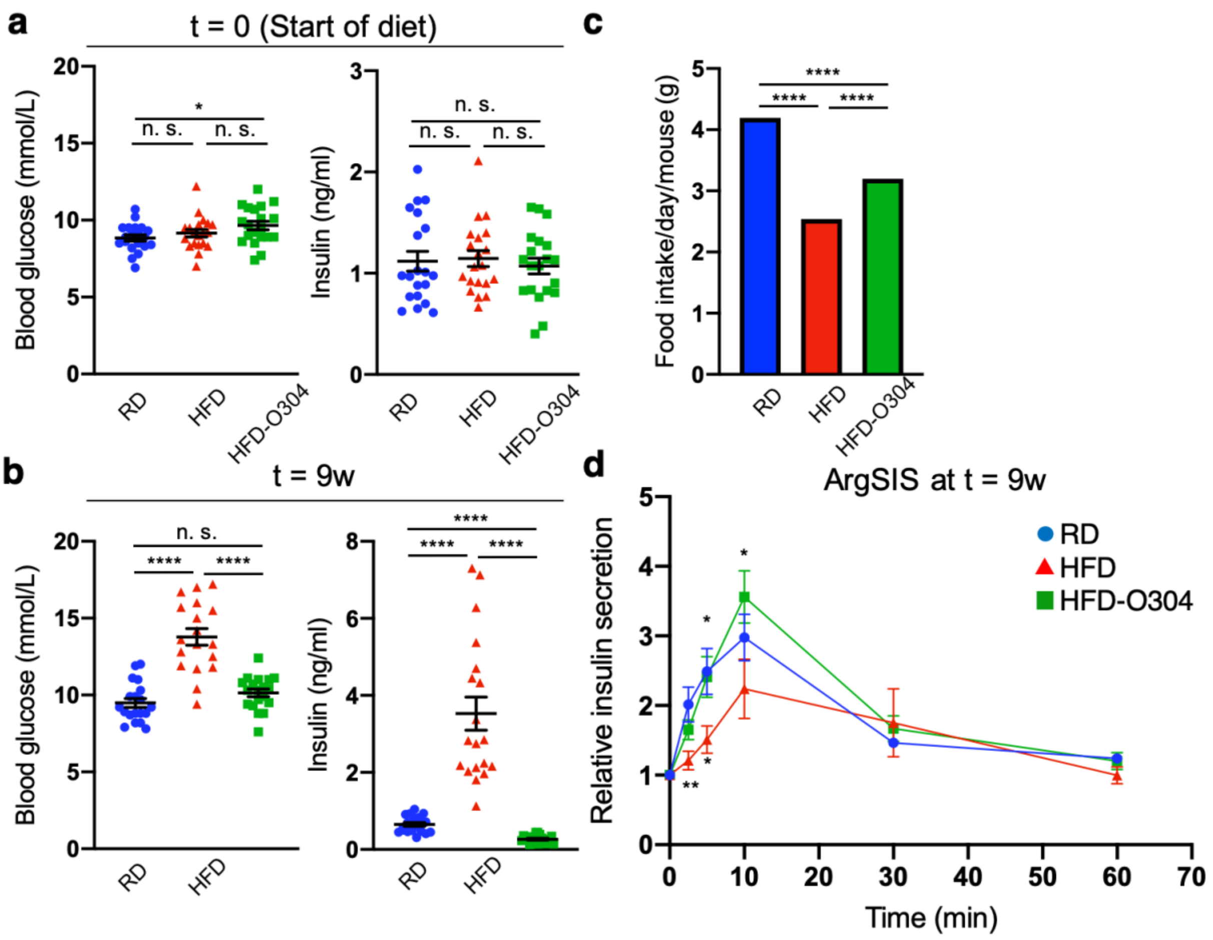

Fig. S1 O304 protects mice from developing impaired blood glucose homeostasis and insulin secretion on a HFD. (a) Fasted blood glucose and insulin for RD, HFD and HFD-O304 mice at start of diet (n=20 for all conditions) and **(b)** after 9 weeks of diet (n=20 for RD and HFD-O304, n=19 for HFD). (c) **(d)** Arginine stimulated insulin secretion test (ArgSIS) after 9 weeks of diet for RD (n=8), HFD (n=10), HFD-O304 (n=10) mice. X-axis reflects time in minutes after administration of arginine (1mg/g body weight). Asterix above lines indicate significance between HFD and HFD-O304, asterix below lines indicate significance between RD and HFD. There are no significant differences between RD and HFD-O304. *p<0.05, **p<0.01, ****p<0.0001, n. s. = not significant (Student's t-test).

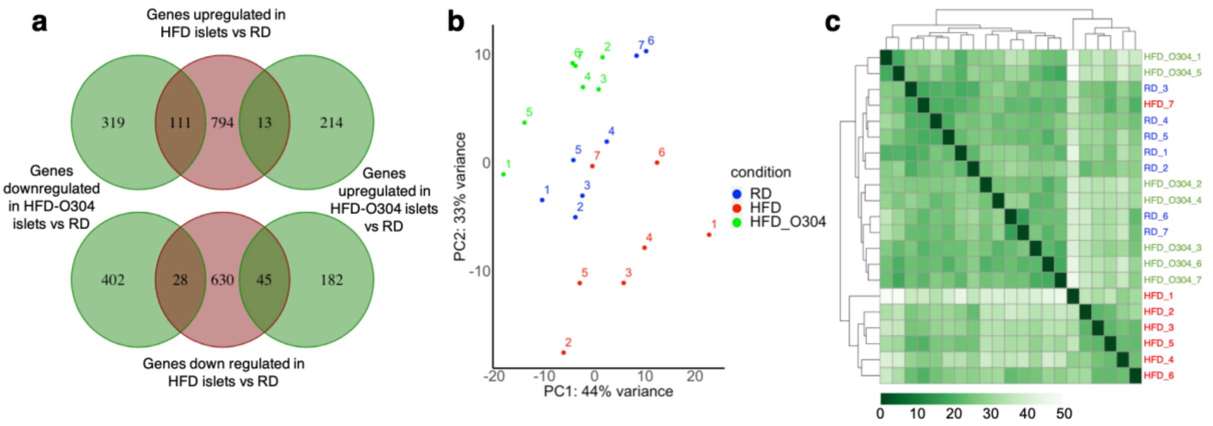

Fig. S2 O304 prevents transcriptional changes in islets of Langerhans induced by HFD. (a) Venn diagram of overlap between genes upregulated (upper panel) or downregulated (lower panel) in HFD islets compared to differentially expressed genes (DEGs) in HFD-O304 islets. **(b)** PCA plot of RNA-seq data. **(c)** Hierarchical clustering and heatmap based on RNA-seq data from RD, HFD and HFD-O304 islets.

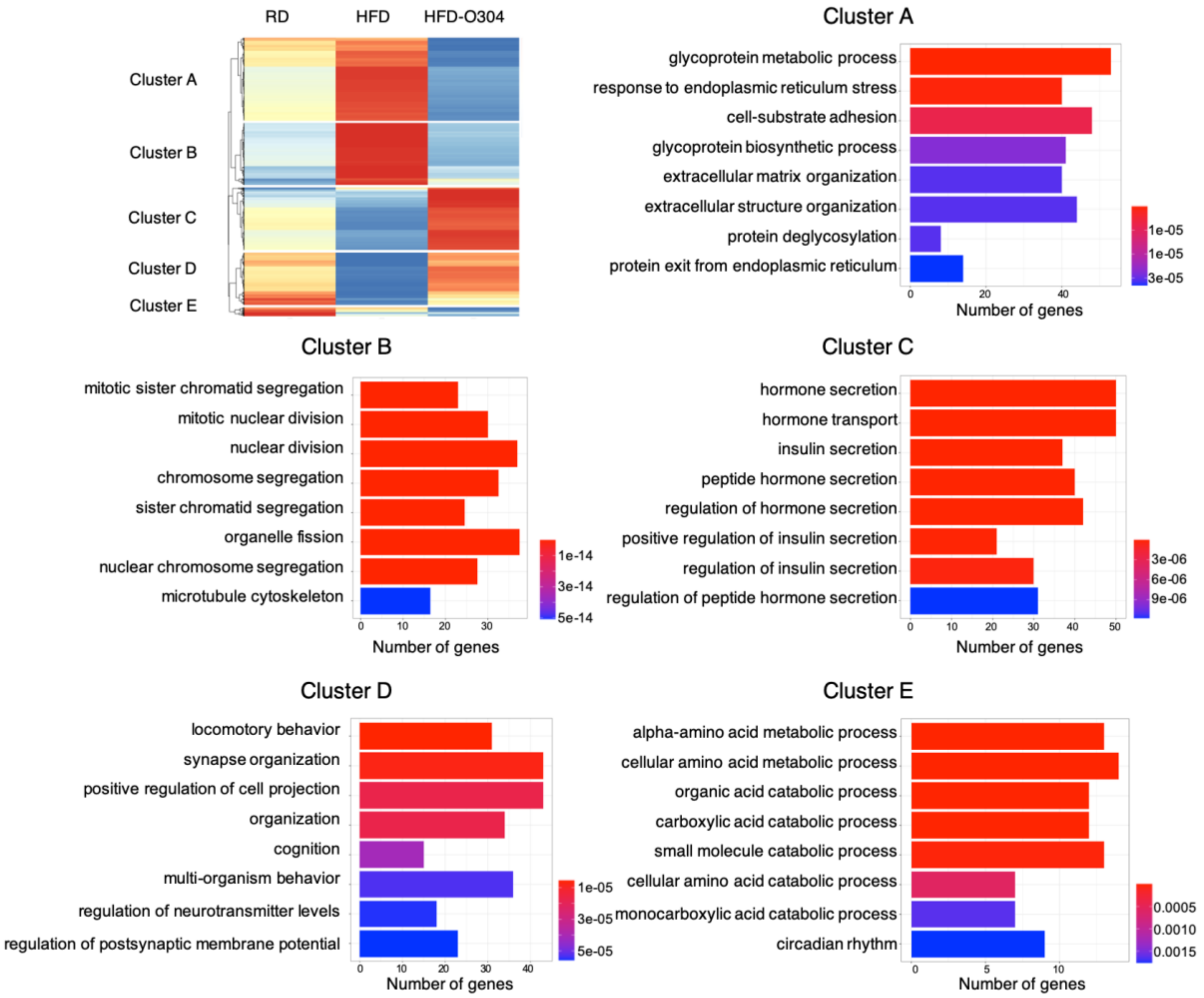

Fig. S3 GO term enrichment analysis indicate that O304 induces gene expression changes related to β–cell function. Top 8 GO terms enriched in each of the group of differentially expressed genes as defined by K-means clustering (left upper panel). Genes in Cluster A and B are more abundantly expressed in HFD islets and are related to ER stress and protein processing, and cell proliferation, respectively. Genes in cluster C and D are less abundantly expressed in HFD islets and relate to insulin and hormone secretion, and neuronal function, respectively. Cluster E genes are most abundantly expressed in RD islets and least in HFD-O304 islets and relate to amino acid metabolism and other catabolic processes. Bar color indicates adjusted p-value (FDR < 0.01) as indicated on scale on right side of each barchart.

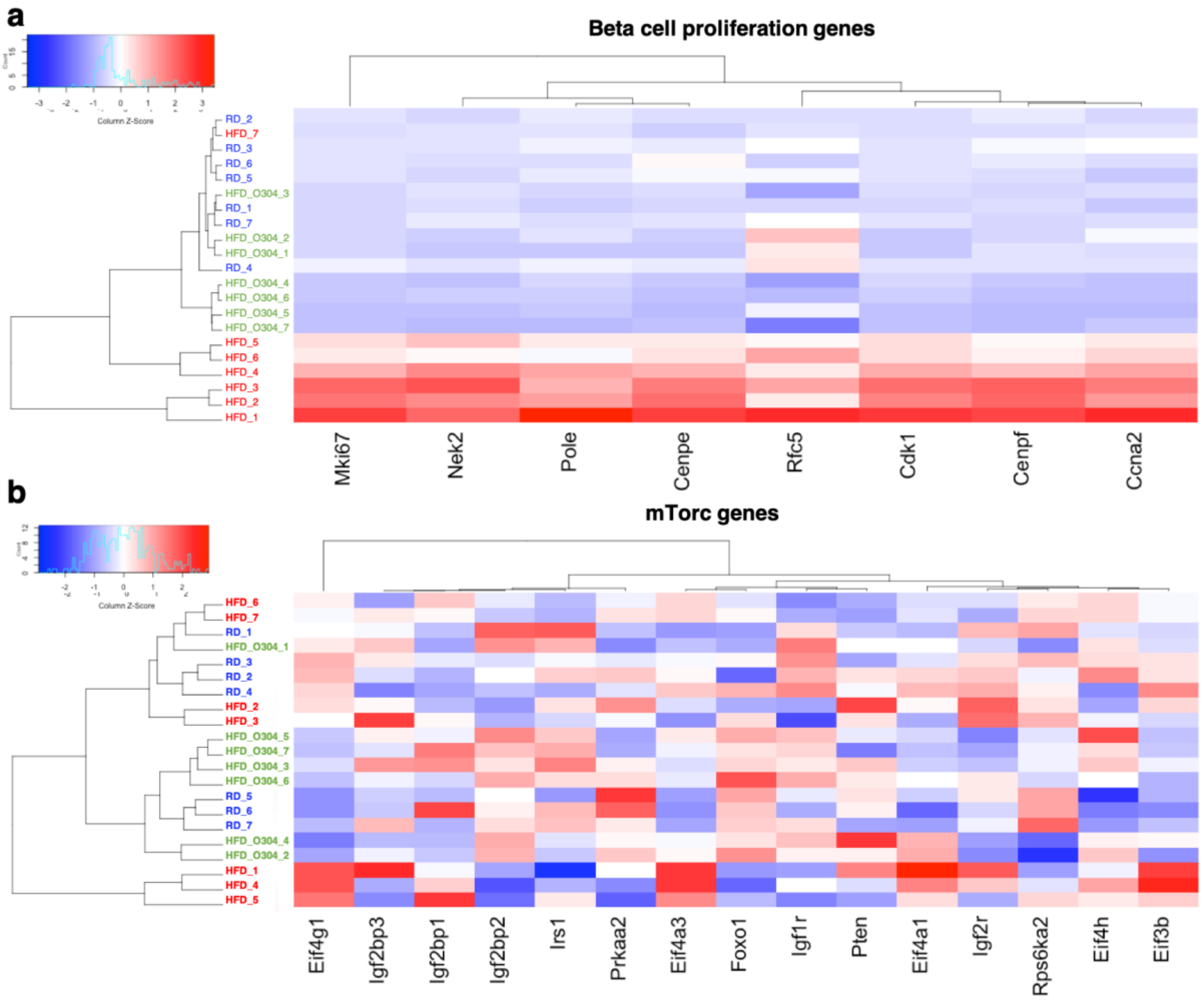

Fig. S4 O304-mediated AMPK activation suppresses cell proliferation in pancreatic islets. Hierarchical clustering and heatmap of expression levels for genes associated with **(a)** β-cell proliferation and **(b)** mTorc signalling. Genes for RD (blue) , HFD (red) and HFD-0304 islets (green).

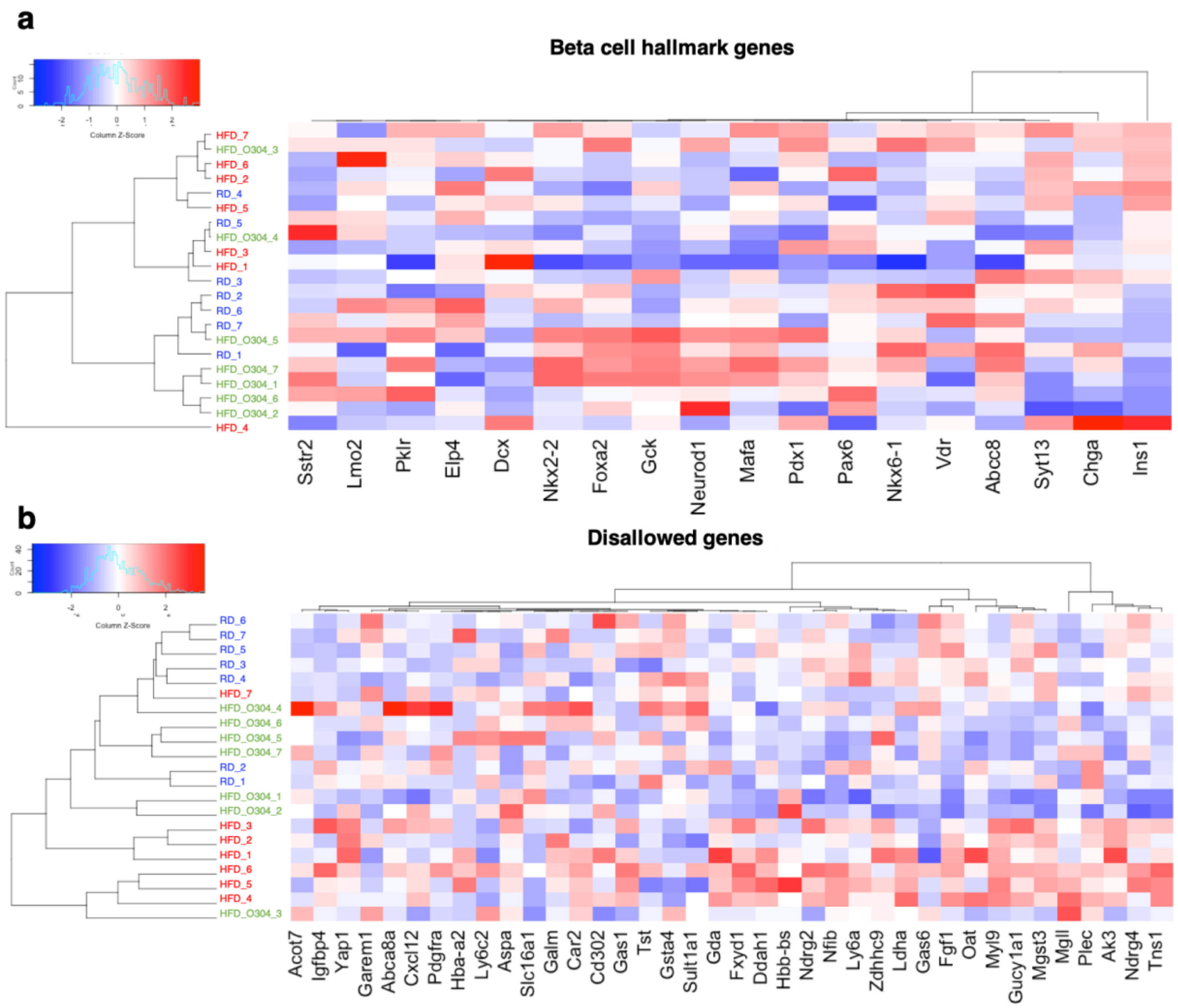

Fig. S5 HFD does not induce major expression changes in β-cell hallmark genes nor in genes normally repressed in β-cells. Hierarchical clustering and heatmap of expression levels for **(a)** β-cell hallmark genes and (**b)** disallowed β-cell genes for RD (blue) , HFD (red) and HFD-0304 islets (green).

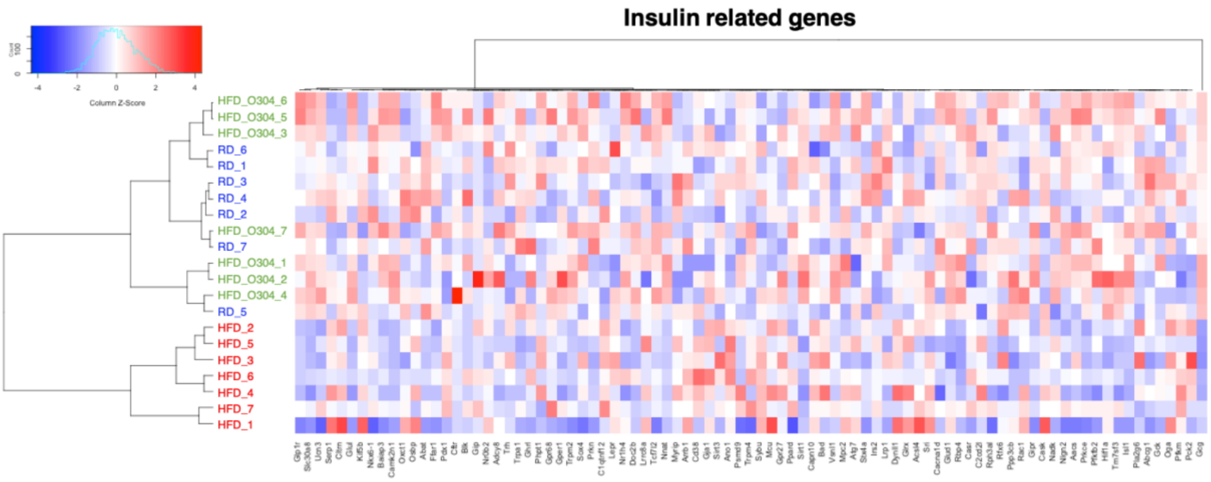

Fig. S6 Expression of genes involved in insulin secretion is normalised in O304-treated islets. Hierarchical clustering and heatmap of expression levels for genes associated with insulin secretion (derived form GO:0035774 and GO:0032024) for RD (blue letters) , HFD (red letters) and HFD-0304 islets (green letters).

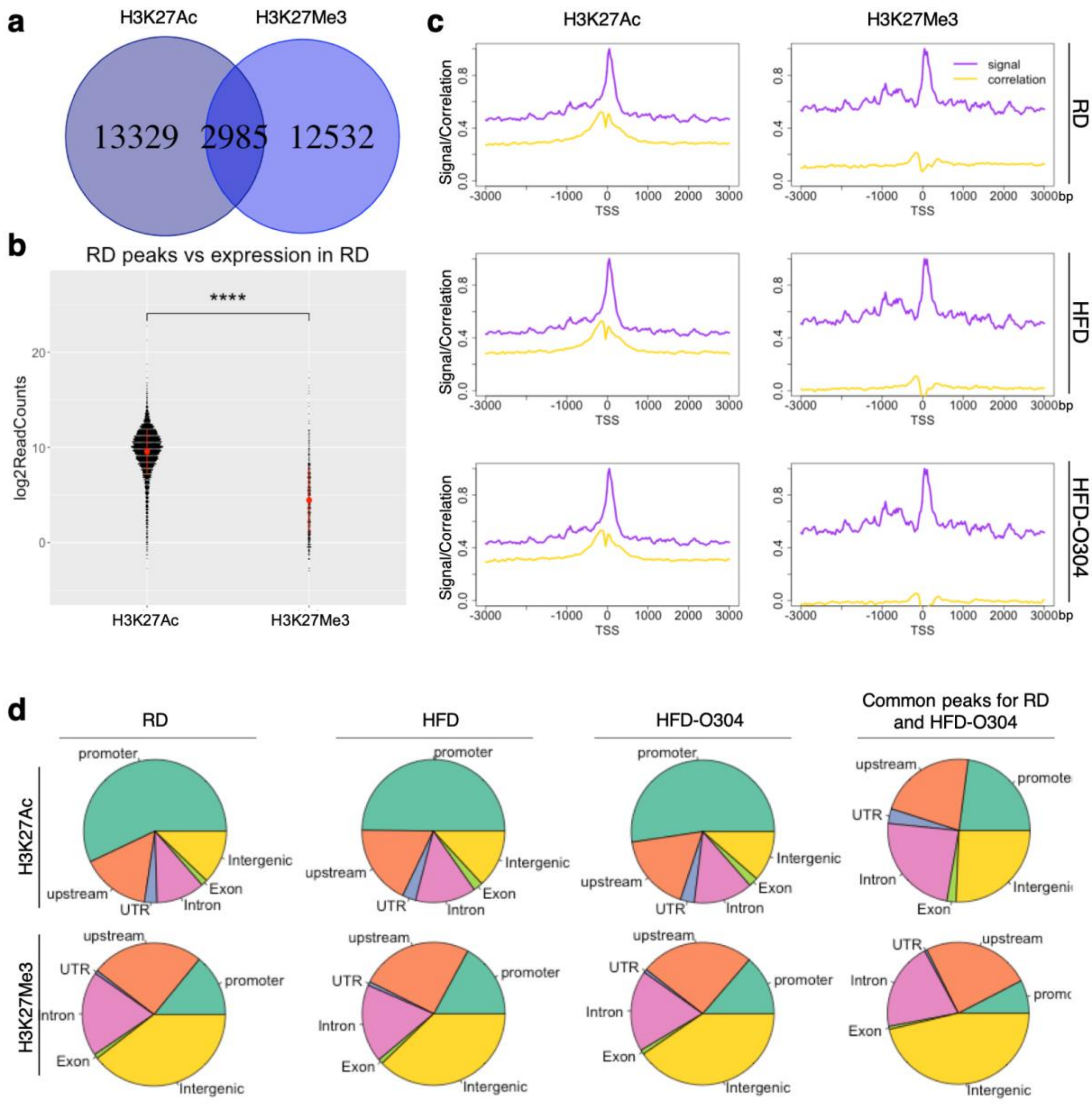

Fig. S7 Correlation between ChIP-seq and RNA-seq data. (a) Venn diagram showing the minor overlap between called H3K27Ac and H3K27Me3 binding regions in RD islets. **(b)** Expression of genes in the proximity of H3K27Ac and H3K27Me3 binding sites, showing that that H3K27Ac associates with highly expressed genes. (**c**) Correlation (yellow line) between mean ChIP-seq signal (purple line) and gene expression levels for H3K27Ac (left column) and H3K27Me3 (right column) in RD, HFD, HFD-0304 islets. **(d)** Proportion of genomic context for ChIP-seq peaks in RD-, HFD-, HFD-O304-islets, as well as for peaks shared between RD- and HFD-O304-islets. Upper row: H3K27Ac, lower row: H3K27Me3. Promoters are defined

as 1kb upstream of transcriptional start site (TSS) while upstream regions are defined as 1-10kb upstream of TSS.

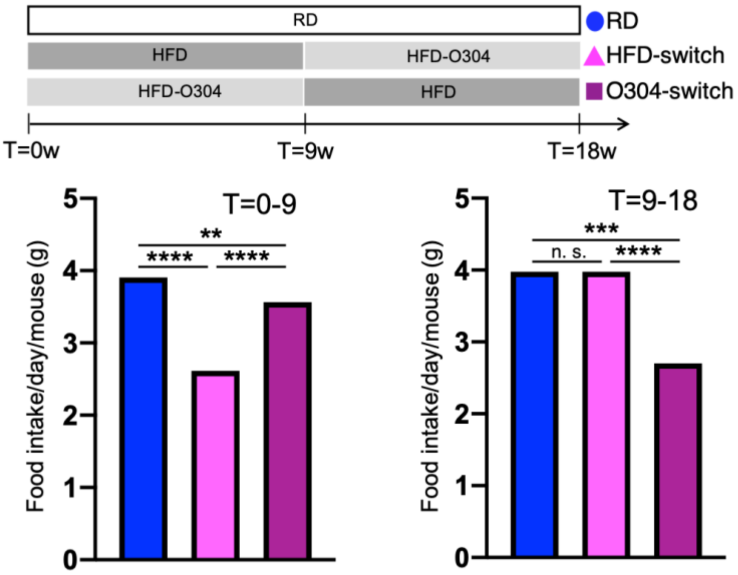

Fig. S8 Increased food intake in O304 treated CBA mice. (a) Average food intake of RD, HFD-switch and O304-switch mice before diet switch and after diet switch as indicated. **p<0.01, ****p<0.0001, n. s. = not significant (Student's t-test).

Table S1 Differentially expressed genes between RD and HFD or HFD-O304. (See supplementary excel file "Supplementary_table_S1")

Table S2 GO and KEGG term enrichment for clusters of differentially expressed gene. (See supplementary excel file "Supplementary_table_S2")

Table S3 Oligo sequences used for qRT-PCR. (See supplementary excel file "Supplementary_table_S3")
